# The doubling effect of COVID-19 cases on key health indicators

**DOI:** 10.1371/journal.pone.0275523

**Published:** 2022-11-23

**Authors:** Oana Petrof, Maxime Fajgenblat, Thomas Neyens, Geert Molenberghs, Christel Faes

**Affiliations:** 1 I-Biostat, DSI, Hasselt University, Diepenbeek, Belgium; 2 Laboratory of Freshwater Ecology, Evolution and Conservation, KU Leuven, Leuven, Belgium; 3 Leuven Biostatistics and Statistical Bioinformatics Centre (L-BioStat), KU Leuven, Leuven, Belgium; 4 I-BioStat, KU Leuven University of Leuven, Leuven, Belgium; Lagos State University, NIGERIA

## Abstract

From the beginning of the COVID-19 pandemic, researchers advised policy makers to make informed decisions towards the adoption of mitigating interventions. Key easy-to-interpret metrics applied over time can measure the public health impact of epidemic outbreaks. We propose a novel method which quantifies the effect of hospitalizations or mortality when the number of COVID-19 cases doubles. Two analyses are used, a country-by-country analysis and a multi-country approach which considers all countries simultaneously. The new measure is applied to several European countries, where the presence of different variants, vaccination rates and intervention measures taken over time leads to a different risk. Based on our results, the vaccination campaign has a clear effect for all countries analyzed, reducing the risk over time. However, the constant emergence of new variants combined with distinct intervention measures impacts differently the risk per country.

## Introduction

Doubling of a key metric, the time span over which it occurs, and its impact on other metrics, are important concepts in life sciences and epidemiology [1]. The doubling time has been well studied and applied to, for example, infectious disease epidemics, population size, tumor growth, and in vitro cell growth [2]. The dynamics of infectious disease outbreaks are commonly measured by the doubling time, especially during the initial epidemic stage, and used to quantify the threat posed by infectious disease outbreaks [3–6]. In this paper, a relatively straightforward but useful measure is proposed to quantify the public health threat of an epidemic, denoted as the “doubling effect”, which mathematically takes the form of a relative risk. The doubling effect quantifies the epidemiological impact on hospitalizations/mortality when the number of cases/hospitalizations doubles. For instance, if the doubling effect is 1.5, this implies that when the number of cases doubles, the hospitalizations will increase by 50%. This is of great interest for policy makers, since an increase of 50% in the number of hospitalizations or mortality can be considered against the background of resources available, and hence it can be gauged whether such an effect can be accommodated or rather represents an strong burden for the health care system of a country.

Given that over 346 million confirmed COVID-19 cases were recorded worldwide, with more than 5.5 million confirmed deaths as of 23 January 2022 [7], policy makers need guidance to make informed decisions towards the adoption of mitigating interventions. Easy-to-understand metrics that can easily be applied and evaluated over time, are therefore fundamental to measure the public health impact of epidemic outbreaks.

Being an RNA virus, SARS-CoV-2 exhibits fundamentally different characteristics compared to DNA viruses [8]. In particular, mutation rates of RNA viruses are generally orders of magnitudes higher, as their RNA polymerases lack the proofreading abilities of the DNA polymerases involved in the replication of DNA viruses [9, 10]. While this feature played less over the first half year of the pandemic, it changed from early September 2020 onwards, when the first variants turned up [11]. In several cases, new waves were provoked at least in part by emerging variants, which changed not only the transmissibility but often also the pathogenicity of the virus [8]. In this paper, we study the Alpha variant, first detected in November 2020 in the United Kingdom [8, 12], the Beta variant, first detected in January 2021 in South Africa, [12], the Delta variant first detected in March 2021 in India [13], and the Omicron variant, first detected in South Africa in November 2021 [14]. From 2021 onwards, the emergence and spread of variants of concern took place against the background of COVID-19 vaccination in the countries involved, altering the evolution of all metrics involved relative to what it would have been had no vaccination taken place.

The purpose of this paper is to propose a model to estimate the epidemiological impact of doubling of cases during an epidemic and to provide an assessment of the relationship between the metrics’ evolution in several countries within the light of surges of new variants. We study the effect of doubling of cases on key metrics, with the emphasis on hospitalizations and mortality in the context of the COVID-19 pandemic.

## Data selection

The data used in this study are retrieved from the Our World in Data (OWID) website [15]. OWID collects official data from government sources, dedicated government institutions, research articles, and international institutions or statistical agencies [15] for a large number of countries worldwide. The data are updated daily.

We include data from North-Western European countries (United Kingdom, Belgium, the Netherlands, Denmark, Norway), Central-Southern European countries (Spain, France, Germany), and Eastern European countries (Estonia, the Czech Republic, Latvia, Croatia). The selection is based on the availability of data. On 24 November 2021, South Africa was the first country to detect and report the Omicron variant of the SARS-CoV-2 virus [16]. This new variant led to an unprecedented surge in cases, showing to be the most transmissible variant of the virus seen thus far [16]. Within days, several countries in Europe reported new positive cases with the new Omicron variant [17]. For this, we choose to include South Africa in our analysis [18].

We use data representing the newly confirmed COVID-19 cases, the newly patients admitted to hospitals, and new deaths attributed to COVID-19.

The analysis is performed over a 19-month period, from September 1st, 2020 until March 31st, 2022. The earliest COVID-19 waves are not included due to the very limited testing (capacity) during the initial phases of the pandemic [19]. All analysis are performed at a weekly level, eliminating day-of-the-week effects.

## Materials and methods

In the first analysis, we model the relationship between the number of newly confirmed COVID-19 cases and new hospitalizations. We repeat the same analysis for the following scenarios: the effect of doubling COVID-19 positive cases on mortality, as well as the effect of doubling hospitalization on mortality.

### Base doubling model

Let *Y*_*t*_ be the weekly sum of confirmed number of cases at time *t* (i.e., the sum of the cases at *t* − 6, *t* − 5 … *t*) and *H*_*t*_ the weekly sum of newly hospitalized patients at time *t* (i.e., the sum of the hospitalized patients at *t* − 6, *t* − 5 … *t*). A negative binomial regression is used to model the number of hospitalizations:

$$\begin{array}{ccc} H_{t} & ∼ & \text{NegBin}(\mu_{t},\phi ), \end{array}$$ (1)

where *NegBin*(*μ*_*t*_, *ϕ*) represents the negative binomial distribution with mean *μ*_*t*_ and overdispersion parameter *ϕ*. This model explicitly accounts for the overdispersion of the count data commonly observed in epidemic outbreak data. The doubling model describes the relationship between cases and hospitalizations as:

$$\begin{array}{ccc} \text{log}(\mu_{t}) & = & \beta {\text{log}}_{2}\left(Y_{t-\delta }\right), \end{array}$$ (2)

where *δ* denotes a delay (lag) time amongst the impact of cases on hospitalisations, log(.) is the natural logarithmic function and log_2_(.) is the logarithmic function with base 2. First note that relationship (2) between the mean number of new hospital admissions and the cases can be rewritten as:

$$\begin{array}{ccc} \mu_{t} & = & \text{exp}\{\beta {\text{log}}_{2}\left(Y_{t-\delta }\right)\}\\ & = & \text{exp}\{\beta \frac{\text{log}(Y_{t-\delta })}{\text{log}(2)}\}\\ & = & \text{exp}\{\text{log}(Y_{t-\delta }^{\beta /\text{log}(2)})\}\\ & = & Y_{t-\delta }^{\beta /\text{log}(2)}, \end{array}$$

establishing that this model assumes a power-function relationship between the hospitalisations and cases. Secondly, note that when the number of cases doubles, the term log_2_(*Y*) increases by a single unit, i.e., log_2_(2*Y*) = log_2_(*Y*) + 1; and as a result the effect of doubling in the number of cases is that the expected number of hospitalizations increases with the multiplicative factor exp(*β*), which we denote as the “doubling effect”. The latter interpretation is particularly useful for policy makers who can use it to assess the current situation in their country and compare it to the evolution of other countries, such as neighboring countries for example, given that multiple countries are included in our study. Policy makers can evaluate such an effect against the background of their country’s stringency, vaccination rate, and circulation of variants, for example, relative to that in other countries. These factors are not included in the model itself, to retain its simplicity of interpretation.

### Time-varying doubling model

We further extend model (1) and (2), to accommodate non-linear time-varying effects through the inclusion of B-splines [20–22], thus allowing flexible time-varying relationships [23]. Knots are placed at several places within the data range, to identify the points where adjacent functional pieces join [23]. The number of knots can either be pre-specified or chosen in a data-driven manner. Both options are investigated (see Section 3.4).

A varying coefficient model [24], also called locally parametric model, is linear in regressors, but their coefficients are allowed to vary smoothly with the value of the other variables, which are commonly called effect modifiers. Keeping the linearity of the regressors allows us to maintain the interpretation, for instance, of doubling the number of cases on hospitalizations, as explained above.

The time-varying doubling effect model (with *K* basis functions), extending the base doubling model (2), then takes the form:

$$\begin{array}{ccc} \text{log}(\mu_{t}) & = & {\text{log}}_{2}\left(Y_{t-\delta }\right)\sum_{k=1}^{K}\beta_{k}f_{k}\left(t\right), \end{array}$$ (3)

where *f*_*k*_() is the *k*’th basis function mapping an interaction between each time *t* with the covariate of interest [24]. The component $\sum_{k=1}^{K}\beta_{k}f_{k}\left(t\right)$ is the time varying doubling effect.

### Multi-country time-varying doubling model

Finally, we extend the time-varying doubling model by modelling all considered countries simultaneously:

$$\begin{array}{ccc} H_{t,c} & ∼ & \text{NegBin}(\mu_{t,c},\phi_{c}), \end{array}$$ (4)

$$\begin{array}{ccc} \text{log}(\mu_{t,c}) & = & {\text{log}}_{2}\left(Y_{t-\delta ,c}\right)\sum_{k=1}^{K}\beta_{k,c}f_{k}\left(t\right), \end{array}$$ (5)

where the subscript *c* refers to country-specific variables or parameters. Though all countries are modelled simultaneously, it does not pool information across countries (i.e., no latent interactions between the parameters of individual countries are assumed).

In the following, we refer to the *country-by-country model* to refer to model (3) estimated separately for each country through restricted maximum likelihood (REML), and to the *multi-country model* to refer to model (4) and (5) for the second one, estimated through Markov chain Monte Carlo (MCMC). For each scenario, we visualise the results of both models side-by-side, facilitating comparison. As both models were independently developed and coded by two different authors, comparing their output enables the assessment of their sensitivity to implementation differences.

### Model implementation and fitting

We fit the country-by-country time-varying doubling model for each country and for each scenario separately through REML using the gam-function of the mgcv 1.8.38 package [25] in R v.4.1.2 [26]. We investigate several delays (lags) *δ* and compare them using a model selection criterion. In our implementation, we compare several delay times for every individual country in the country-by-country analysis, and select the model with the lowest AIC value as the best fitting one [27]. The delays investigated for each analysis together with the best fitting model can be seen in Table 1 in the Supporting information section. A dashboard is currently being developed which allows the users to visualize the doubling effects per country using up-to-date data.

**Table 1:** The best fitting delay (lag) *δ* in days chosen via the AIC for both methods used in this study for the country-by-country model. The abbreviations *C-H*, *C-M* and *H-M* refer to the cases to hospitalizations, cases to mortality and hospitalizations to mortality scenarios respectively.

	C-H	C-M	H-M
UK	7	21	14
Denmark	7	14	14
Belgium	7	14	7
Spain	7	21	14
France	7	21	7
Netherlands	7	21	7
Germany	7	21	14
Czech Republic	7	14	7
Norway	14	21	14
Latvia	7	21	7
Croatia	14	14	7
Estonia	14	14	7
South Africa	7	21	14

We implemented this multi-country model for each of the three considered scenarios using the probabilistic programming language Stan, and performed MCMC sampling through the rstan v.2.21.2 package (Stan Development Team, 2020) in R v.4.1.2 [26]. We assume weakly informative standard-normal priors for the *ϕ*_*c*_ and the *β*_*k*,*c*_ parameters. The combined analysis of multi-country data allows for a more fine-grained investigation of the optimal number of knots and lags. Therefore, we considered models with 0 to 21 lags and 5 to 21 knots for each scenario separately. Based on the leave-one-out information criterium (LOOIC; Vehtari et al. 2017), we chose a lag of 5 days and 13 basis functions for the cases-hospitalizations analysis, a lag of 10 days and 15 basis functions for the cases-mortality analysis and a lag of 5 days and 15 basis functions for the hospitalizations-mortality analysis. A full overview of the model selection results is shown in S2 Fig).

## Results

The estimated relative risk corresponding to the doubling effect, is plotted over time as well as against the vaccination rate for all countries. In both analyses, the country-by-country and the multi-country analysis lead to very similar results. S1 Fig shows the effect of doubling cases on hospitalizations. Results for North-Western European countries are presented in the top panel, for Eastern European countries in the middle panel and for Central-Southern European countries and South Africa in the bottom panel. Other scenarios are presented in the Supporting information section (S3 and S4 Figs).

In the North-Western European countries, the United Kingdom has the highest relative risk, starting in September 2020 with a 70% increase in hospitalization if the number of confirmed COVID-19 cases doubled. This risk decreases to 60% in May 2021 and remains stable afterwards. The Nordic countries Norway and Denmark have a lower doubling risk, with approximately a 50% increase in hospitalizations when the number of cases double. The trend of the relative risk in time is not as stable as compared to the one for the United Kingdom, possibly due to the smaller population and a lower number of hospitalizations. Belgium and the Netherlands experience a 50 to 60% increase in hospitalizations due to doubling in the number of cases, with higher relative risks in the first year of the pandemic.

In the Eastern European countries group, Croatia has the largest relative risk, with an increase from 50% up to 70%, while in Estonia the relative risk ranges 40% and 60%. Latvia has a relative risk and evolution similar to Croatia. The Czech Republic has a lower relative risk in June 2021, but experiences a higher one in October 2021.

France and Germany have a similar relative risk over the entire time period, showing a slow but steady decrease of the relative risk since May 2021. Spain and the United Kingdom are the only two countries that almost reached an 80% increase in the hospitalizations when the COVID-19 cases doubled in the second pandemic wave, October/December 2020.

South Africa has the largest relative risk out of all countries analyzed. Note the peak of the relative risk in November/December 2021, probably due to the highly contagious Omicron variant, which hit the country when the vaccination rate was still low, i.e., around 30% based on the OWID data. The other European countries did not experience this type of increase in the relative risk during the same time period.

We provide the results of the last two scenarios in the Supporting information section (S3 and S4 Figs). For the North-Western European countries, the relative risk profiles look similar with a larger relative risk in January 2021, a decreased risk in May–June 2021, and a slight increase in December 2021. The same can be observed for Croatia, Latvia, the Czech Republic, and Estonia. If in the first analysis, i.e., the cases-hospitalizations scenario, South Africa had a much larger relative risk compared to all the other European countries, for the cases-mortality scenario, South Africa’s profile is subsequently getting closer to the other countries.

Finally, the hospitalization-mortality analysis shows the same trend as the cases-hospitalizations, but the relative risks for all countries seem to be less stable over time. For every country, the number of hospitalizations and deaths are much lower than the case incidence and hospitalizations.

The relative risks plotted over the vaccination rate for all countries are presented in the Supporting information (S5–S7 Figs). The effects of the vaccination campaign can be seen for almost all countries over all scenarios, lowering the relative risk as the percentage of the vaccinated population is increasing. However, during a pandemic, many factors can play a significant role in the evolution and spread of the disease, for instance, non-pharmaceutical interventions. Moreover, as time elapses, there are typically waning effects in the vaccination effectiveness, which are visible for all countries analyzed. The increase in the relative risk in June–July 2021 due to the more pathogenic delta variant, seems to diminish the vaccination effects.

## Discussion

Some limitations of our study need to be addressed. The number of confirmed COVID-19 cases is an undercount of the total number of cases. Especially asymptomatic cases are underrepresented among the confirmed cases [28, 29]. Worldwide surveillance systems tend to include only the more severe cases, whereas mild cases and non-diagnosed infections are excluded [30]. At the same time, gross underreporting of cases in the early phases occurred due to lack of diagnostic tests, healthcare workers, and other resources [3]. While diagnostic tests were in sufficient supply from around May 2020 onward, the testing system of a country was often stressed during peak periods. We assume underreporting is heterogeneous across countries. Next to that, there are different testing regimes and contact-tracing strategies between countries aiming at suppressing the coronavirus transmission [19].

For a given country, similar profiles are seen regardless of the analysis performed. This happens against the background of the fact that every country adopted a different vaccination strategy and had a different vaccination rate, together with varying stringency levels. Next to these, it is worth mentioning that there are cultural differences between countries. However, for all countries across all analyses a decrease in the relative risk in the May–June 2021 period is observed, except for South Africa. This is the moment when vaccination reached around 50% of the population in the West-European countries. Based on S1 Fig, the Eastern-European countries saw an increase in the relative risk in October/November 2021, arguably due to the Delta variant. It should be noted that the metric developed here reflects the past and current epidemiological situation in a country, rather than being developed for prediction purposes. Policy makers can use our method to assess the vulnerability of a country health system. A relative risk higher than in other countries is an alarming situation and shows that it is time for them to take action and find strategies to reduce the risk and impact on the health system. A high relative risk shows that further investigation is needed regarding the vaccination strategy, the possible surge of a new variant, differences in the health systems in terms of hospitalizing patients. One beneficial aspect worth mentioning is the possibility of one country to learn and adapt based on the situation of another country. Next to these advantages, policy makers can gauge the doubling effect against the background of their country’s stringency, vaccination rate, and circulation of variants, for example, relative to that in other countries. These factors are not included in the model itself, to retain its simplicity of interpretation, but it could be considered in future analyses.

It should be noted that though no information is directly pooled across the countries in the multi-country model (e.g. by means of shared parameters), this approach offers four major advantages which complement the country-by-country analysis. First, the multi-country analysis easily enables comparisons across the different countries, either directly within the model or by post-processing the obtained posterior distributions. Second, by considering all data jointly, we were able to perform a detailed investigation on the selection of the optimal lag (cf. S2 Fig), whereas this would be impossible in the country-by-country analysis due to data sparsity. Third, the multi-country analysis offers convenience for the applied user, as the model only needs to be fitted once to obtain inference on all countries. Fourth, the multi-country analysis serves a validation purpose as it has been developed independently and as it features a different inferential method compared to the country-by-country, while still yielding very similar results. In summary, we consider that the multi-country analysis nicely complements the country-by-country analysis. Further capitalizing on the multi-country model by including explanatory variables to understand differences among countries would be a very interesting perspective to develop as further research.

## Conclusion

In this work, we have presented a modeling approach that enables estimation of the epidemiological impact of doubling of cases on such other metrics as hospitalizations and COVID-19 mortality, and at the same time, provide a description of the metrics’ evolution for several countries over time. In a similar manner, the effect of doubling hospitalizations on mortality is examined. This is done in a country-by-country as well as in a multi-country analysis and both analyses lead to very similar results.

For all European countries, the vaccination campaign provided some layer of protection against the Delta and Omicron variants, while for countries with a low vaccination rate, such as the Eastern European countries or South Africa, the relative risk increased regardless of the analysis performed, indicating that the medical system was placed under heavy pressure. In almost all European countries, the vaccination campaign started in December 2020–January 2021, continued during the course of 2021, with several Western European countries reaching around 50% full vaccination rate in June–July 2021. The effect of vaccination can be seen for all countries. It is clear that everywhere the relative risk decreases after June–July 2021. The decrease in risk can also be caused by other factors that play an important role such as the varying stringency measures implemented by all countries and their time-varying nature. Indeed, a disease outbreak is influenced by multiple factors such as severity that shapes the transmission network structure [31] as well as the fraction of the susceptible population and the effects of behavioral changes and non-pharmaceutical interventions [5, 31–33].

## Supporting information

S1 Fig Analysis of cases-hospitalizations relationship, based on the country-by-country model (left) and on the multi-country model (right), using one week, ten days and two weeks delay. The relative risk, exp(*β*), together with a 95% confidence interval for the time-varying doubling model and exponent of the posterior mean with a 95% equal tail credibility interval for the multi-country time-varying doubling model.
(EPS)

S2 Fig Values of the leave-one-out information criterium (LOOIC) for models differing in the chosen lag and number of basis functions, for each of the three scenarios. Lighter colors indicate a lower LOOIC and, hence, correspond to an improved model performance compared to darker colors.
(EPS)

S3 Fig Analysis of the cases-mortality based on the country-by-country model (left) and on the multi-country model (right), using one week, ten days and two weeks delay. The doubling effect together with a 95% confidence interval for the time-varying doubling model and exponent of the posterior mean with a the 95% equal tail credibility interval for the multi-country time-varying doubling model.
(EPS)

S4 Fig Analysis of the hospitalization-mortality based on the country-by-country model (left) and on the multi-country model (right), using one week, ten days and two weeks delay. The doubling effect together with a 95% confidence interval for the time-varying doubling model and exponent of the posterior mean with a the 95% equal tail credibility interval for the multi-country time-varying doubling model.
(EPS)

S5 Fig The doubling effect together with a 95% confidence interval for the country-by-country model over the vaccination rate for the cases-hospitalization scenario. (EPS)

S6 Fig The doubling effect together with a 95% confidence interval for the country-by-country model over the vaccination rate for the cases-mortality scenario. (EPS)

S7 Fig The doubling effect together with a 95% confidence interval for the country-by-country model over the vaccination rate for the hospitalization-mortality scenario. (EPS)
